# Supplementary material for: All-optical control of high-purity trions in nanoscale waveguide
Source: Nat Commun. 2023 Apr 12;14:1891. doi: 10.1038/s41467-023-37481-1 (PMC10097695; doi:10.1038/s41467-023-37481-1)
Supplement: Supplementary file 1 — Supplementary Information [file 41467_2023_37481_MOESM1_ESM.pdf]

Supplementary Information

All-optical control of high-purity trions in nanoscale waveguide

Hyeongwoo Lee,[1] Yeonjeong Koo,[1] Shailabh Kumar,[2,3] Yunjo Jeong,[4] Dong Gwon Heo,[5] Soo Ho Choi,[6] Huitae Joo,[1] Mingu Kang,[1] Radwanul Hasan Siddique,[2,3] Ki Kang Kim,[6,7] Hong Seok Lee,[5] Sangmin An,[5] Hyuck Choo*,[2,8] and Kyoung-Duck Park*[1]

[1]*Department of Physics, Pohang University of Science and Technology (POSTECH), Pohang 37673, Republic of Korea*

[2]*Department of Medical Engineering, California Institute of Technology (Caltech), CA 91125, USA*

[3]*Meta Vision Lab, Samsung Advanced Institute of Technology (SAIT), CA 91101, USA*

[4]Institute of Advanced Composite Materials, Korea Institute of Science and Technology, Jeonbuk 55324, Republic of Korea

[5]*Department of Physics, Research Institute of Physics and Chemistry, Jeonbuk National University, Jeonju 54896, Republic of Korea*

[6]*Center for Integrated Nanostructure Physics, Institute for Basic Science (IBS), Suwon 16419, Republic of Korea*

[7]*Department of Energy Science, Sungkyunkwan University (SKKU), Suwon 16419, Republic of Korea*

[8]*Advanced Sensor Lab, Device Research Center, Samsung Advanced Institute of Technology (SAIT), Suwon 16678, Republic of Korea*

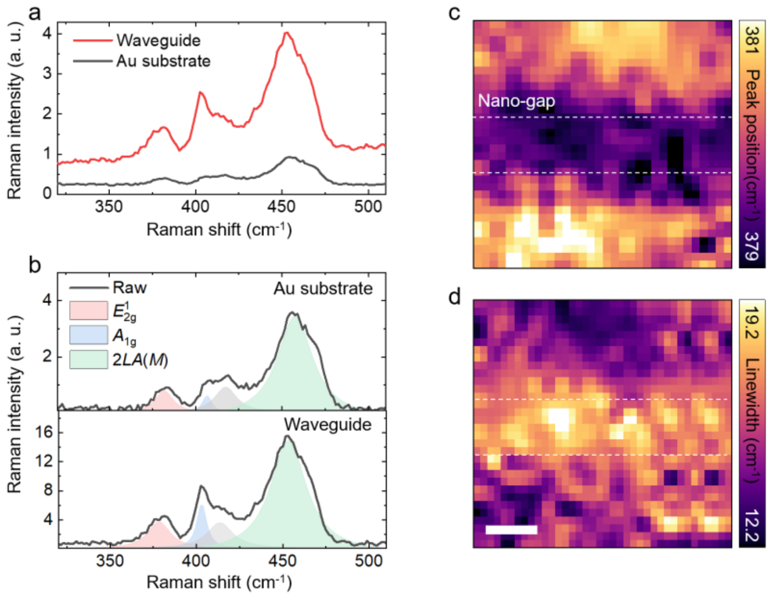

Supplementary Fig. 1. Raman scattering characteristics of the suspended MoS_2 ML. (a) Raman spectra of MoS_2 ML transferred on Au substrate (black) and suspended on trionic waveguide (red). (b) Lorentz fitted Raman spectra of the MoS_2 ML transferred on Au substrate (top) and suspended on trionic waveguide (bottom). Red, blue, and green filled peaks indicate E^1_{2g}, A_{1g}, and 2*LA*(*M*) Raman modes. Peak position image (c) and linewidth image (d) for E^1_{2g} mode Raman spectra. Scale bar is 400 nm.

In order to induce the inhomogeneous strain on the ultrathin trionic waveguide device, the formation of the well-suspended MoS_2 ML on the device is important. We confirm the well-suspended MoS_2 ML by comparing the Raman spectra measured at the Au substrate and the trionic waveguide, as shown in Supplementary Fig. 1a-b [1]. When the MoS_2 ML is suspended, both E^1_{2g} and A_{1g} mode are redshifted with noticeable spectral broadening. Supplementary Fig. 1c-d show the redshifted peak position with the spectral broadening at the nanogap region, which are in good agreement with the previous studies [2, 3].

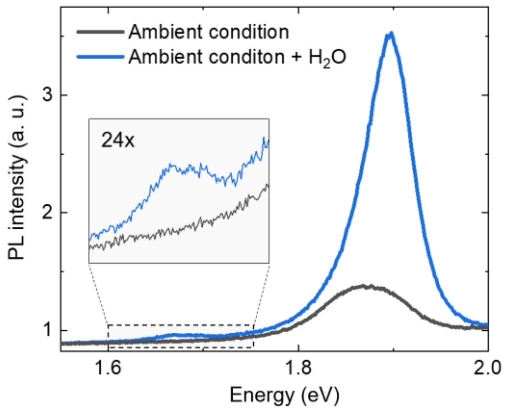

Supplementary Fig. 2. PL spectra of MoS_2 ML under ambient condition without (black) and with H_2O molecules (blue) on crystal surface. Zoomed in plot indicate Raman spectra of H_2O molecule.

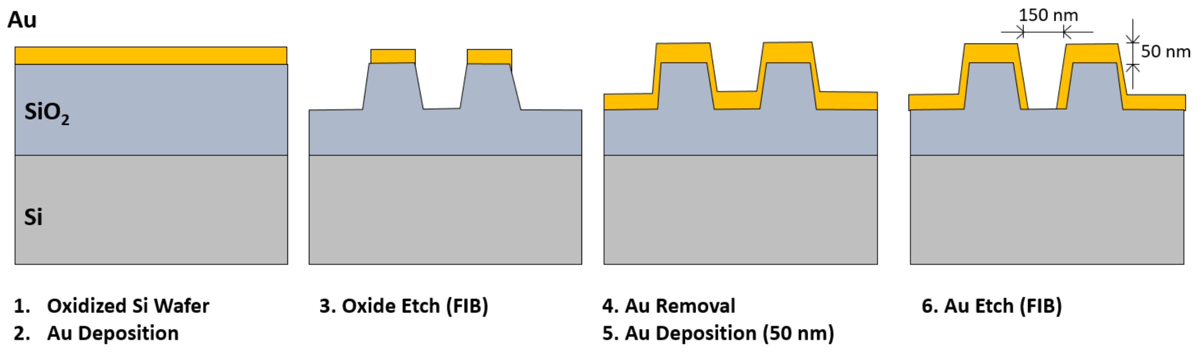

Supplementary Fig. 3. Fabrication process of lateral MIM waveguide.

The coupling of light and SPP propagation rely on a lateral MIM geometry where the top and sidewalls of the nanogap are gold-coated, whereas bottom of the channel is SiO_2. For fabrication of the channel, we start with a SOI wafer, and deposit 150 nm of Au on it. Then we perform a FIB milling step to etch into the SiO_2. As seen in the Supplementary Fig. 3, after milling the resultant nanogaps have a thin layer of gold on top, but much of the sidewall is SiO_2. This milling process results in a slight taper to the sidewalls, which is crucial for subsequent gold deposition step. The top layer of gold is then removed using a gold etchant. A fresh layer of Au (50 nm) is deposited using e-beam, and coats the top, sidewalls, and bottom of the nanogap. A second round of milling is then performed to remove the gold from bottom of the nanogap channel. As the figure illustrates, the first milling process is an oxide etch step, whereas the second milling is an Au etch step to remove gold from bottom of the channels.

The parameters for fabricating lateral MIM waveguide, such as gap size and height, are optimized in the way of maximizing the efficiency of SPP coupling to the nanogap while also minimizing losses during SPP propagation in the nanogap. Specifically, the nanogap lateral MIM geometry (Au-SiO_2-Au) has been designed to provide a high contrast between the effective refractive index inside the channel and the refractive index of the substrate ($nSiO_2$ = 1.46) providing a higher coupling efficiency. Furthermore, coupling of incoming laser light with lateral MIM nanogap to activate SPP mode proceeds through scattering from the edges and sidewalls of the gap. This method where light is coupled to Au-SiO_2-Au MIM nanogap through edge/tail-end illumination has been discussed in detail in previous works [4, 5, 6].

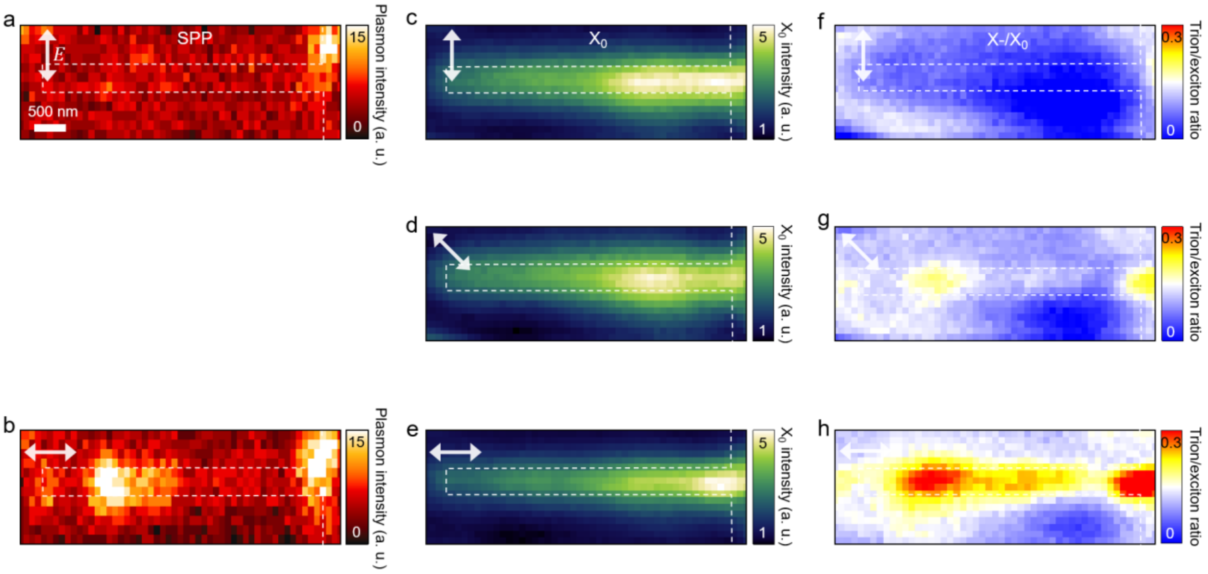

Supplementary Fig. 4. 2D image of Fig. 2 in the main text. SPP images with excitation polarization across (a) and along (b) waveguide. (c-e) X_0 PL images of with different excitation polarizations. (f-h) PL images of X-/X_0 ratio with different excitation polarizations. White dashed line is guideline for waveguide structure based on Supplementary Fig. 5.

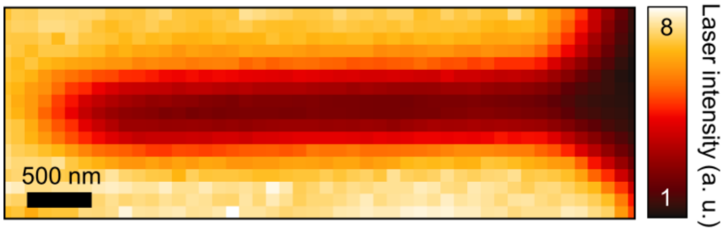

Supplementary Fig. 5. Rayleigh scattering image of the trionic waveguide. Guideline for scan area in Fig. 2 is based on this Rayleigh scattering image.

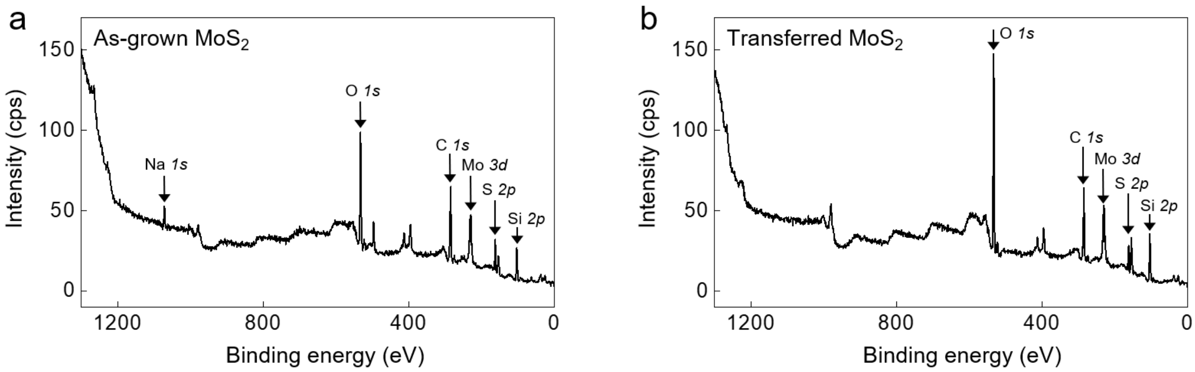

Supplementary Fig. 6. XPS spectra of as-grown (a) and transferred (b) MoS_2 monolayer.

To exclude the defect-related electron generation, we perform X-ray photoelectron spectroscopy (XPS) before and after transferring MoS_2 monolayer onto the target substrate. As a result, we obtain Mo:S ratio of 1:1.976 for as-grown MoS_2 monolayer (Supplementary Fig. 6a) while 1:1.998 for transferred MoS_2 monolayer (Supplementary Fig. 6b). After the transfer of MoS2 onto the substrate, the Mo:S ratio is highly close to 1:2, indicating negligible generation of defects during the transfer. Note that slightly dominant detection of Mo can be attributed to the Molybdenum precursor.

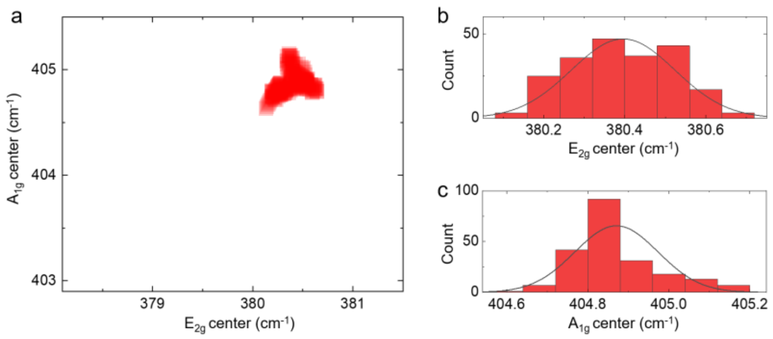

Supplementary Fig. 7. Charge density distribution in MoS_2 ML transferred onto Au substrate. (a) A_{1g} center versus E_{2g} center measured at various spots. Probability histogram of E_{2g} center (b) and A_{1g} center (c).

To investigate the charge distribution of MoS_2 ML, we obtain Raman spectra at various spots and extract the peak position of E_{2g} and A_{1g} peak, as shown in Supplementary Fig. 7a. Supplementary Fig. 7b-c shows the probability distribution of E_{2g} and A_{1g} peak, respectively. The linewidth of the fitted probability distribution exhibits ~0.2 cm^{-1}, demonstrating the homogeneity of the charge distribution [7, 8].

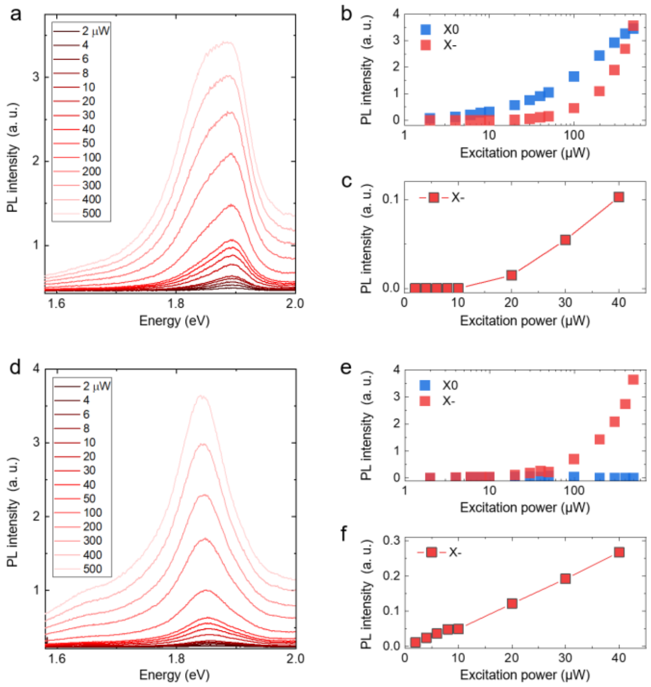

Supplementary Fig. 8. Power dependence of exciton-to-trion conversion. (a) Excitation-power-dependent PL spectra at Au substrate. (b) Extracted X_0 intensity (blue) and X- intensity (red). (c) Extracted X- intensity at low excitation powers. (d) Excitation-power-dependent PL spectra at SPP mode. (e) Extracted X_0 intensity (blue) and X- intensity (red). (f) Extracted X- intensity at low excitation powers.

To confirm the role of SPP mode on the exciton-to-trion conversion, while excluding the effect of defect-related provision of electrons, we measure excitation-power-dependent PL spectra at Au substrate and SPP mode. At Au substrate, the linewidth of the PL spectra increases as increasing the excitation power, attributed to the emerging trion peak, as shown in Supplementary Fig. 8a. In contrast to gradually increased X_0 intensity as a function of excitation power, the X- intensity shows negligible changes at the low excitation power (<10 μW), which is possibly due to the inactivation of defect-induced electrons at low excitation power (Supplementary Fig. 8b-c) [9]. On the other hand, the dominant X- peak are continuously observed with increasing excitation power at the SPP mode, as shown in Supplementary Fig. 8d. Correspondingly, the X- intensity linearly increases as increasing excitation power even at the low excitation power, as shown in Supplementary Fig. 8e-f. This behavior well indicates the plasmon-induced hot electron injection and consequently increased X- intensity with high exciton-to-trion conversion efficiency [10].

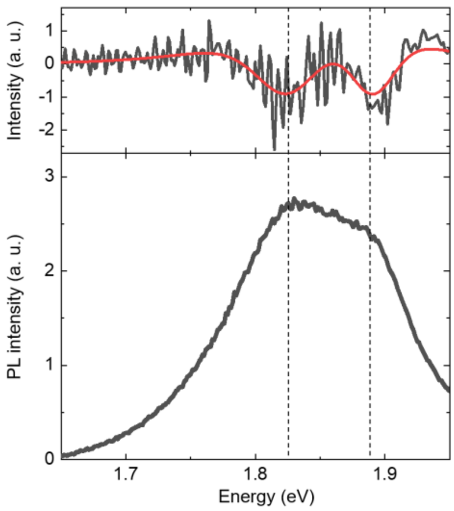

Supplementary Fig. 9. Second derivative curve (top) obtained from PL spectrum in Fig. 3d, with excitation polarization of 45º (bottom). Two minima in second derivative are assigned to neutral exciton peak and trion peak.

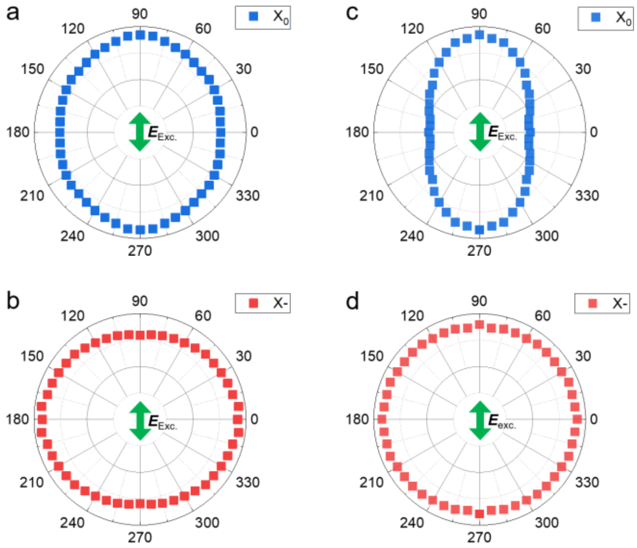

Supplementary Fig. 10. Polarization degree of excitons and trions. Polar plot for intensity of X_0 (a) and X- (b) as function of detection angle at Au substrate. Polar plot for intensity of X_0 (c) and X- (d) as function of detection angle at SPP mode.

To investigate the strain-induced optical characteristics of X_0 and X- emission, we obtain PL intensities of X_0 and X- with changing detection angle. At Au substrate, both X_0 and X- emissions exhibits negligible polarization degree (Supplementary Fig. 10a-b), in good agreement with previous study [11]. By contrast, X_0 emission shows the noticeable polarization degree of ~0.52 (Supplementary Fig. 10c) in contrast to still negligible polarization degree of X- emission (Supplementary Fig. 10d), attributed to the SPP-induced excitation of additional X_0 [12, 13, 14]. Note that SPP mode is excited with the vertical excitation polarization, which corresponds to waveguide axis.

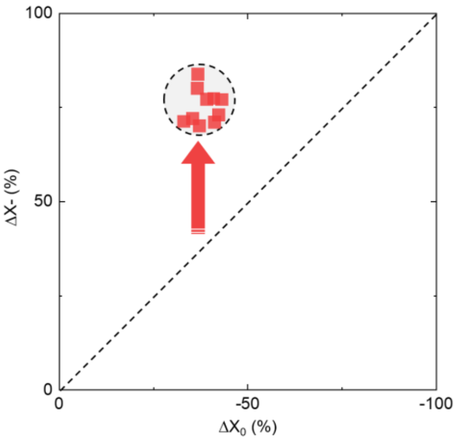

Supplementary Fig. 11. Change in trion density (ΔX-) as function of change in neutral exciton density (ΔX-), with activation of SPP mode.

To investigate the possible energy transfer between the excitons and the SPP, we obtain the PL spectra with and without the SPP mode. We then extract X_0 and X- intensities by fitting PL spectra with Lorentz function. Supplementary Fig. 11 shows the change of trion density (ΔX-) as a function of the change of neutral exciton density (ΔX_0). Without the influence of the SPP, decrease in X_0 density should correspond to increase in X- density with dominating highly efficient exciton-to-trion conversion process at strain gradient geometry. However, Supplementary Fig. 11 exhibits the extra increase in the trion density. This is probably due to the SPP-induced excitation of excitons [13, 14] and these additionally generated excitons are converted to trions as enough number of electrons are confined at the center of nanogap.

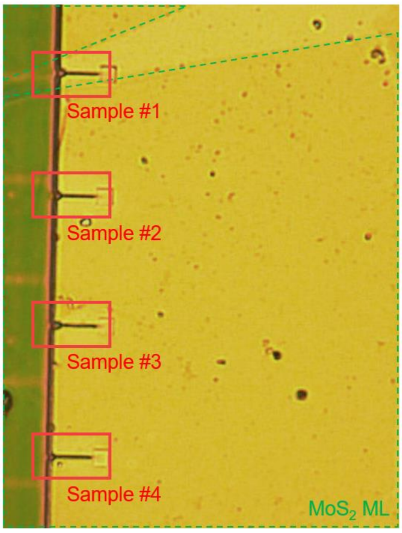

Supplementary Fig. 12. Optical microscope image of lateral MIM waveguide device. MoS_2 ML is transferred on the waveguides #2-4 excepting the waveguide #1.

We fabricate four identical lateral MIM waveguide structure (sample #1-4). The MoS_2 ML is transferred on the three lateral MIM waveguides (sample #2-4) while sample #1 remains bare. As shown in Fig. 4c-d, SPP signal is spectrally overlapped with MoS2 PL. Therefore, to find the optical phase mask with the SPP signal without the influence of MoS_2 PL, we used the waveguide #1. Because all lateral MIM waveguides are identical, they have identical SPP mode and share an optimal phase mask. Therefore, we optimize the phase mask at the device #1 and then move onto the device #2 to perform the main experiments.

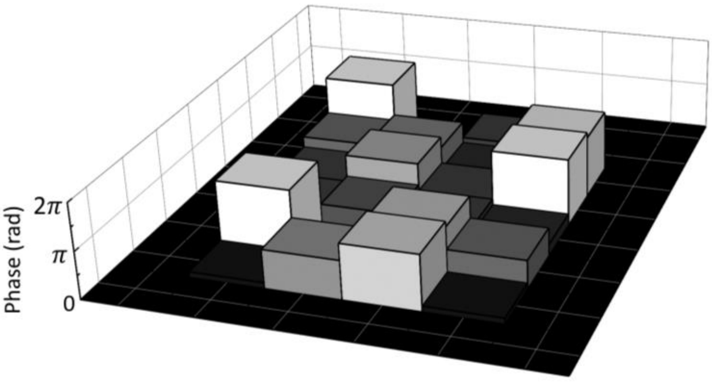

Supplementary Fig. 13. Optimal phase mask of Fig. 4 in main text.

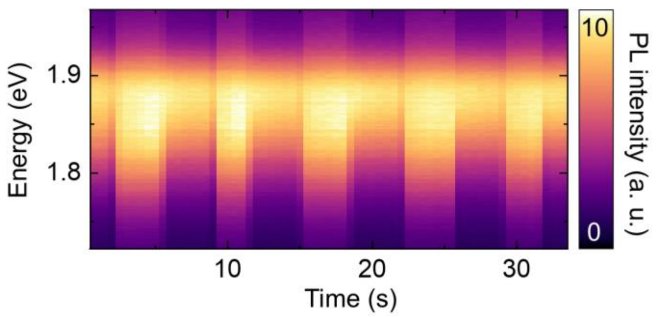

Supplementary Fig. 14. On/off switching of exciton-to-trion conversion. Time-series PL spectra during on/off switching of exciton-to-trion conversion, before normalization of Fig. 4e.

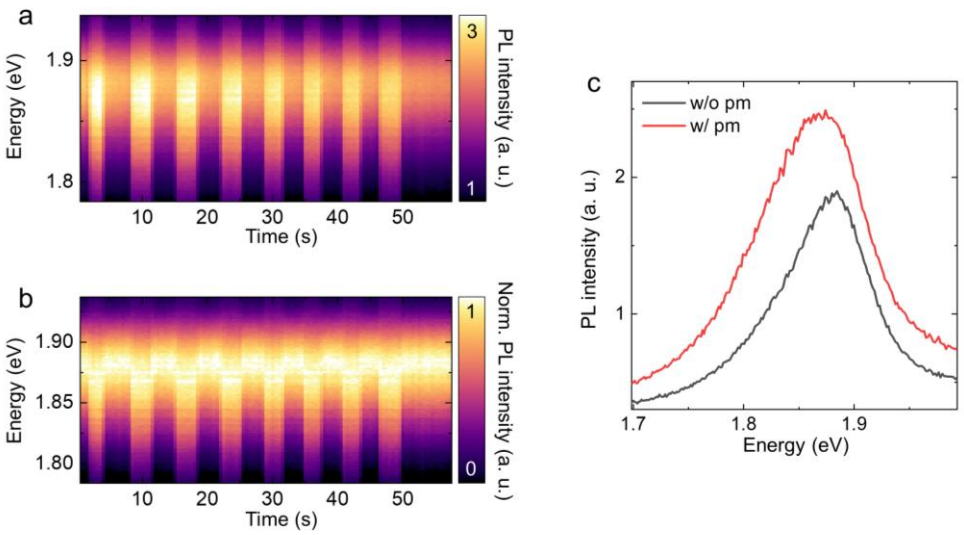

Supplementary Fig. 15. Reproducibility of modulating exciton-to-trion conversion location. (a) Time-series PL response during on/off switching of exciton-to-trion conversion. (b) Normalization of (a). (c) Representative PL spectra with (red) and without (black) optimal phase mask.

The controllability and reproducibility are confirmed by conducting a wavefront shaping at the different weak SPP region. Supplementary Fig. 15a shows the dynamic switching between X_0 dominant emission and X- dominant emission with the phase mask optimized for the current location. The normalized spectral image in Supplementary Fig. 15b and the representative PL spectra in Supplementary Fig. 15c show the distinct transition between X_0 dominant and X- dominant emissions.

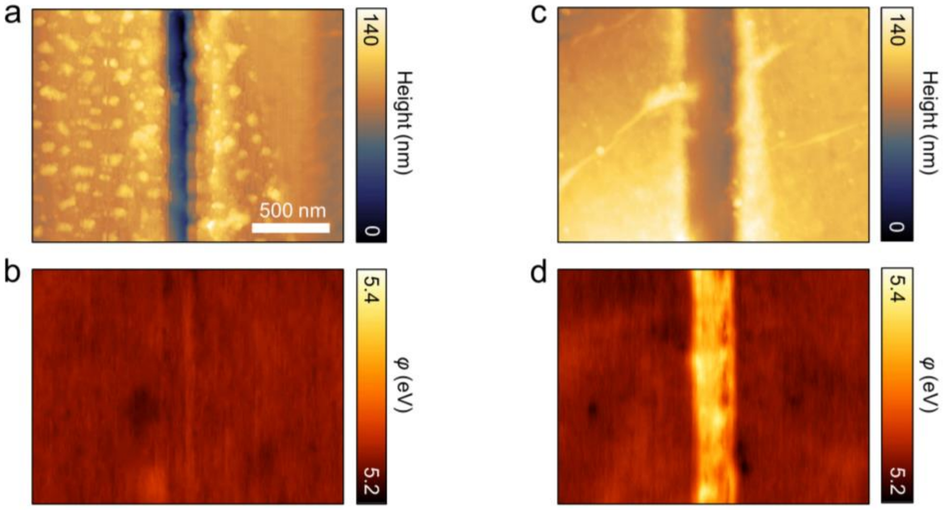

Supplementary Fig. 16. Work function of MoS_2 ML on nanogap of trionic waveguide. Topography image (a) and corresponding work function image (b) of nanogap without MoS_2 ML. Topography image (c) and corresponding work function image (d) of suspended MoS_2 ML on nanogap.

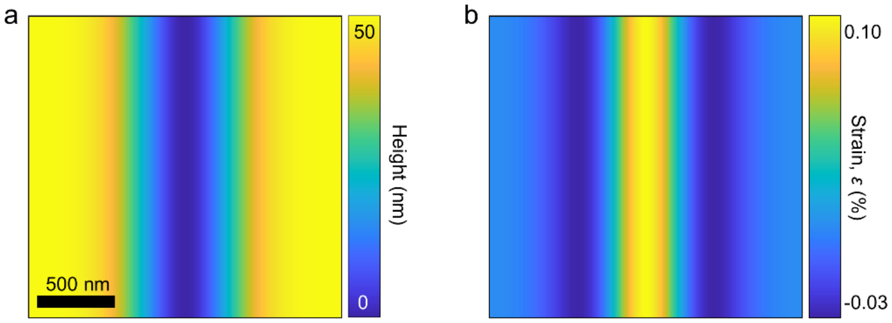

Supplementary Fig. 17. Estimation of the strain profile. (a) Image of fitted line shape function based on experimentally measured topography profile of MoS_2 ML on nanogap of ultrathin trionic waveguide. (b) Corresponding strain image estimated by continuum theory for a thin and elastic plate [15].

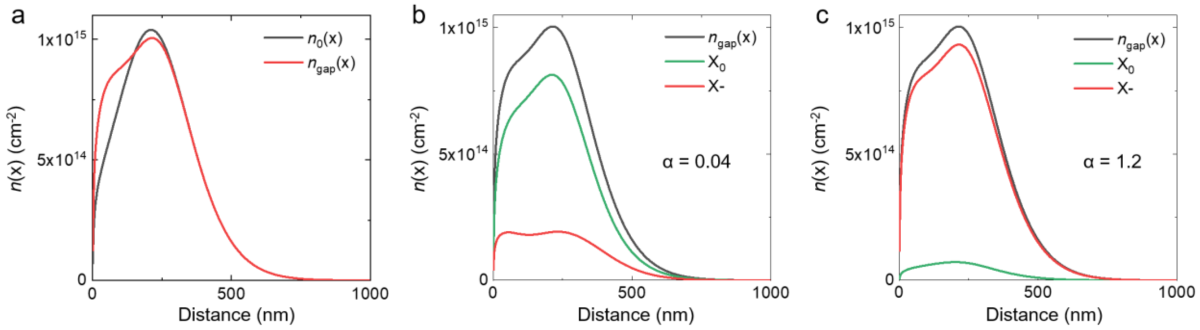

Supplementary Fig. 18. Estimation of spatial distribution of X_0 and X-. (a) Spatial distribution of the photoexcited excitons before considering mass action model with (red) and without (black) strain gradient. Contribution of X_0 (green) and X- (red) in total photoexcited exciton density (black) for α = 0.04 (b) and α = 1.2 (c).

As mentioned in the main text, we subtract the photoexcited exciton density obtained without the strain gradient (black, in Supplementary Fig. 18a) from the exciton density with the strain gradient (red, in Supplementary Fig. 18a) to exclude the effect of the optical excitation. At this stage, we exclude the contribution of X- to clearly investigate the role of strain gradient by the nanogap. Then, we adopt the mass action model to estimate the density ratio of X_0 and X- depending on the background electron density α, as shown in Supplementary Fig. 18b and S18c.

Supplementary References

[1] Fan, J.-H. *et al*. Resonance Raman scattering in bulk 2H-MX_2 (M = Mo, W; X = S, Se) and monolayer MoS_2. *J. Appl. Phys.* **115**, 053527 (2014).

[2] O'Brien, M. *et al.* Raman spectroscopy of suspended MoS_2. *Phys. Status Solidi B* **254**, 1700218 (2017).

[3] Yu, Y. *et al.* Engineering substrate interactions for high luminescence efficiency of transition-metal dichalcogenide monolayers. *Adv. Funct. Mater.* **26**, 4733-4739 (2016).

[4] Choo, H. *et al.* Nanofocusing in a metal–insulator–metal gap plasmon waveguide with a three-dimensional linear taper. *Nat. Photon.* **6**, 838 (2012).

[5] Bao, W. *et al.* Mapping local charge recombination heterogeneity by multidimensional nanospectroscopic imaging. *Science* **338**, 1317 (2012).

[6] Kumar, S. *et al.* Overcoming evanescent field decay using 3D-tapered nanocavities for on-chip targeted molecular analysis. *Nat. Commun.* **11**, 2930 (2020).

[7] Mouri, S. *et al.* Tunable Photoluminescence of Monolayer MoS_2 via Chemical Doping. *Nano Lett.* **13**, 5944 (2013).

[8] Andleeb, S. *et al.* Chemical doping of MoS_2 multilayer by p-toluene sulfonic acid. *Sci. Technol. Adv. Mater.* **16,** 035009 (2016).

[9] Tanoh, A. O. A. *et al.* Enhancing Photoluminescence and Mobilities in WS_2 Monolayers with Oleic Acid Ligands. *Nano Lett.* **19**, 6299 (2019).

[10] Harats, M. G. *et al.* Dynamics and efficient conversion of excitons to trions in non-uniformly strained monolayer WS_2. *Nat. Photon.* **14**, 324 (2020).

[11] Ee, H.-S. *et al.* Tunable Metasurface and Flat Optical Zoom Lens on a Stretchable Substrate. *Laser Photonics Rev.* **16**, 2200008 (2022).

[12] Ming, T. *et al.* Strong Polarization Dependence of Plasmon-Enhanced Fluorescence on Single Gold Nanorods. *Nano Lett.* **9**, 3896 (2009).

[13] Goodfellow, K. M. *et al.* Integrated nanophotonics based on nanowire plasmons and atomically thin material. *Optica* **1**, 149 (2014).

[14] Goodfellow, K. M. *et al.* Direct On-Chip Optical Plasmon Detection with an Atomically Thin Semiconductor. *Nano Lett.* **15**, 5477 (2015).

[15] Landau, L. D. & Lifshitz, E. Course of theoretical physics.
